# Deep Fertilization Enhances Crude Protein Content in Forage Maize by Modulating Key Enzymes of Protein Synthesis Across Plant Organs in Semi-Arid Regions of China

**DOI:** 10.3390/biology14050535

**Published:** 2025-05-12

**Authors:** Hongli Wang, Guoping Zhang, Sicun Yang, Mingsheng Ma, Yanjie Fang, Huizhi Hou, Kangning Lei, Jiade Yin

**Affiliations:** 1 Institute of Dryland Agricultural Sciences, Gansu Academy of Agricultural Science, Lanzhou 730070, China; zhanggp8210@163.com (G.Z.); mamingsh@163.com (M.M.); fangyj82@126.com (Y.F.); houhuizhi666@163.com (H.H.); lkn409@163.com (K.L.); gsyinjiade@163.com (J.Y.); 2 Key Laboratory of Efficient Utilization of Water in Dry Farming of Gansu Province, Lanzhou 730070, China; 3 Key Laboratory of Low-Carbon Green Agriculture in Northwestern China, Ministry of Agriculture and Rural Affairs, Lanzhou 730070, China

**Keywords:** fertilization depth, layered deep fertilization, forage maize, crude protein synthesis, enzyme activity, crude protein content, forage quality

## Abstract

Appropriate fertilization depth promotes the absorption and transport of nutrients, crop growth and yield. However, little is known about whether deep fertilization improves crude protein synthesis and how to regulate it. A two-year field experiment was conducted with various fertilization depths: (1) conventional fertilization (CF), (2) fertilization application depth at 30 cm (DF), and (3) fertilizer average application at depths of 15 cm and 30 cm (AF). The fertilization rates under all treatments were 300 kg N ha^−1^ nitrogen fertilizer (urea, 46% N), 150 kg P_2_O_5_ ha^−1^ calcium superphosphate (16% P_2_O_5_), and 135 kg K_2_O ha^−1^ potassium sulfate (51% K_2_O). The nitrogen/potassium (N/K) ratio, the activities of nitrate reductase [NR], glutamine synthetase [GS], and glutamic pyruvic transaminase [GPT], crude protein content in leaves, stems, and grains, as well as the relationships among the parameters were explored. The result showed that deep fertilization (DF) significantly improved the N/K ratio. NR activity in DF increased by 26.30%, 35.56%, and 57.30% in leaves, stems, and grains, respectively, when compared to conventional fertilization (CF), and by 54.22%, 43.27%, and 28.44% when compared to average fertilization (AF). GS activity in DF increased by 29.67%, 47.96%, and 47.46% in leaves, stems, and grains when compared to CF, and by 40.05%, 31.51%, and 40.62% when compared to AF. GPT activity in DF was significantly higher than CF and AF in grains, and differences between treatments were significant. Crude protein content was significantly correlated with NR and GS activities in leaves, GPT activity in stems, as well as GS and GPT activities in grains. The crude protein content of leaves and grains in DF was significantly higher than in CF and AF. In conclusion, DF significantly improved crude protein synthesis and increased the crude protein content of forage maize by increasing the whole plant N/K ratio, NR and GS activities in leaves, as well as GS and GPT activities in grains. It is a highly efficient cultivation technology that significantly improves the quality of forage maize.

## Simple Summary

Appropriate fertilization depth promotes the absorption and transport of nutrients, crop growth and yield. However, little is known about whether deep fertilization improves crude protein synthesis and how to regulate it. Our results showed that (1) high protein synthesis key enzyme activities contribute to high crude protein content. (2) Crude protein content was significantly correlated with NR and GS activities in leaves, GPT activity in stems, as well as GS and GPT activities in grains. (3) Deep fertilization (DF) significantly increased the crude protein content of forage maize by increasing the NR and GS activities in leaves, as well as GS and GPT activities in grains.

## 1. Introduction

Crude protein reflects the total amount of nitrogen-containing substances in feed, which is the basis for the evaluation of feed nutritional value and compound feed, and is also a necessary nutrient for animal growth and maintenance of life activities [1]. Increasing the crude protein level of feed can significantly increase the daily weight gain rate, feed–meat ratio and feed–egg ratio of livestock, thereby improving the apparent digestibility of nutrients and feed conversion rate [1,2,3]. However, at present, the crude protein content of forage maize is only 3~5% in the semi-arid areas of China, which cannot meet the needs of high-quality forage in aquaculture. Changing the depth of fertilization can have a major impact on crop growth and development. Appropriate deep application of nitrogen fertilizer can reduce the evaporation and loss of fertilizer caused by surface application, promote crop root development, substantially increase plant leaf area, increase chlorophyll and photosynthetic protein contents, delay leaf senescence rates, improve water and fertilizer utilization efficiency, promote crop shoot growth, and substantially increase crop yields [4,5,6,7,8].

Different application positions of fertilizers affect nutrient migration and transformation in the soil [9], in addition to influencing nutrient migration and accumulation in crops [10], thereby affecting crop growth. A fertilization depth of 15 cm has been shown to considerably increase root length, root density, and root surface area of maize; considerably increase nitrogen uptake by 6.5–11.8%, leaf area index, plant height, stem diameter, and aboveground dry matter; grain yield by 13.9–22.4%; and decrease soil nitrate nitrogen residue in the 200–300 cm layer by 17.9–30.7% [4,11,12,13]. An increase in the fertilization depth to 25 cm has no significant effect on the agronomic traits of summer maize, and a further increase to 35 cm has a negative effect on the agronomic traits of summer maize [12]. Some studies have shown that deep fertilizer application to approximately 25 cm of soil layer increases the plant height and stem diameter of maize, promotes the accumulation and transport of dry matter, and substantially increases maize yield [14]. Appropriate fertilization depth increases dry matter accumulation in the aboveground parts of wheat and considerably enhances dry matter accumulation after anthesis. The number of grains per spike, 1000-grain weight, and yield of wheat have been shown to considerably increase when the depth of single-layer strip application of base fertilizer is increased from 8 cm to 24 cm [15,16]. Studies on potatoes have shown that fertilization at 30 cm increases the SPAD index value of chlorophyll content of potato leaves during starch accumulation, promotes water uptake after flowering and potato reproductive growth, substantially increases the number of tubers per plant, tuber weight per plant, and the proportion of large and medium tubers, and increases tuber yield by 77–56.9% [17].

Layered fertilization refers to the application of fertilizer at different soil depths, which is more appropriate for the spatial distribution of nutrients and ensures that nutrients are moderately moved downward through the soil. Layered fertilization can alter the ‘upper fat and lower thin’ phenomenon caused by conventional fertilization application methods, increase nutrient content in the 20–40 cm soil layer [18], promote root system development in lower soil layers, delay root and leaf senescence, and promote crop growth and development [19]. Furthermore, layered fertilization can considerably increase crop water uptake, leaf chlorophyll content (SPAD index), leaf area index, biomass accumulation, crop yield, and fertilizer use efficiency [19,20,21,22].

Current research on layered and deep fertilizer application has focused on crop growth and development, yield, fertilizer utilization, and residual loss. However, few studies have investigated the regulatory mechanisms of crop quality, particularly crude protein synthesis. For forage maize, the crude protein content of the whole plant is a key factor influencing silage making and animal feeding [23]. However, the impact and regulation mechanisms of chemical fertilizer placement on crude protein synthesis in different organs of forage maize under double ridges and furrows with plastic mulching conditions were few reported. In this study, the effects of different fertilization depths on the N/K ratio, enzyme activity, and crude protein content of forage maize were investigated. In addition, the correlation between enzyme activity and crude protein content in different forage maize organs was determined. The physiological regulatory mechanisms of crude protein synthesis were elucidated at the organ level from the perspective of ‘cultivation measures–plant N/K–enzyme activity–crude protein content’. The results of this study form a theoretical basis for the improvement of forage maize quality and promote the adjustment of the crop planting structure in semiarid areas.

## 2. Materials and Methods

### 2.1. Experimental Site

The study site was located at the Dingxi Experimental Station at the Gansu Academy of Agricultural Sciences, Anding District, Dingxi, Gansu Province, China, in the northwest Loess Plateau (104°36′ E, 35°35′ N), which has an altitude of approximately 1970 M ASL. The soil water content (*w*/*w*) at field capacity and wilting point of the study site are 23% and 7.2%, respectively. Based on 35-year rainfall data records (1986–2020), the mean annual rainfall is 415 mm, with nearly 68% occurring between June and September. The relative variability of annual rainfall is 24%, and the mean annual temperature is 6.2 °C. Average annual sunshine hours are 2500 h. The soil is light loam of loess origin without salinity and alkalinity problems. The soil nutrient statuses, namely soil organic matter, total nitrogen (N), total phosphorus, total potassium, ammonium-N, nitrate-N, available phosphorus, and available potassium, were 11.99 g kg^−1^, 1.16 g kg^−1^, 0.73 g kg^−1^, 17.28 g kg^−1^, 4.8 mg kg^−1^, 0.8 mg kg^−1^, 8.66 mg kg^−1^ and 121.50 mg kg^−1^, respectively.

### 2.2. Experimental Design

Three treatments were applied during the growing seasons from 2019 to 2020. All treatments used double ridges and furrows with plastic mulching. One density was set at a hectare of 75,000 plants ha^−1^, with three treatments: (1) conventional fertilization (CF), (2) fertilization application depth at 30 cm (DF), and (3) fertilizer average application at depths of 15 cm and 30 cm (AF).

**Figure 1:**
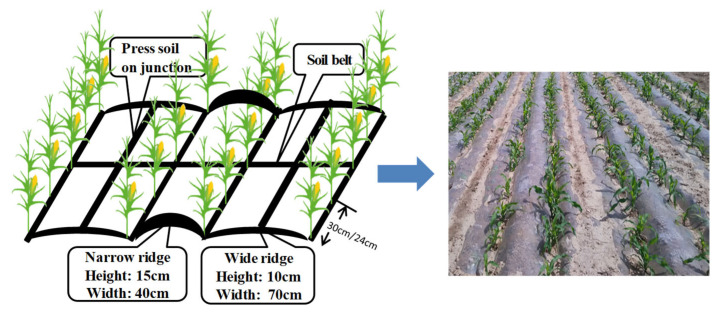
Schematic diagram of the double ridges and furrows with plastic mulching.

The double ridges and furrows with plastic mulching comprised a wide ridge (10 cm high and 70 cm wide) and a narrow ridge (15 cm high and 40 cm wide), and both ridges were mulched with plastic films (Figure 1).

The fertilization rates under all treatments were 300 kg N ha^−1^ nitrogen fertilizer (urea, 46% N), 150 kg P_2_O_5_ ha^−1^ calcium superphosphate (16% P_2_O_5_), and 135 kg K_2_O ha^−1^ potassium sulfate (51% K_2_O). The fertilization method was fertilizer sprinkled on the surface, rotary tillage in CF, and strip fertilization, and the test soil was loosened to 40 cm with a subsoiler (1S-220; Nonghaha Agricultural Machinery Group Co., Ltd., Shijiazhuang, China) before fertilization in DF and CF. The furrow was manually opened to the required depth at the small ridge strip, the soil was backfilled into the furrow after sprinkling the fertilizer, and ridging and film mulching were finally performed. Maize was sown in the furrows. The experimental design was a randomized complete block (PCBD) with four replications. The area of each plot was 63 m^2^ (7 m × 9 m). Maize was sown at a depth of 5–8 cm in the furrows with a soil water content of 10–15% in the 0–30 cm soil layer using a maize planter. The spacing was 24 cm and the planting density was 75,000 plants ha^−1^. Maize was sown on 20 April and harvested at the dough stage of forage maize each year.

### 2.3. Field Sampling

At the jointing, tasseling, filling, and dough stages, three representative plants were selected from each plot, and the leaves were rolled up after removing the veins, the 5–8 cm stems were cut, and the middle part of the maize ear was taken. Samples were wrapped in tin paper, respectively, quickly frozen in liquid nitrogen, and brought back to the laboratory for testing.

### 2.4. Sampling and Measurements

Plant total N and potassium content measurements: Forage corn plants were initially dried at 105 °C for 30 min, then dried at 80 °C to a constant weight and crushed. Thereafter, the crushed samples were passed through a 40-mesh sieve. Total N content was determined using the Kjeldahl method, and total K content was determined using a flame photometer (M425; Beijing Zhongke Keer Instrument Co., Ltd., Beijing, China).

Measurement of nitrate reductase (NR) activity: First, 1.0 g of fresh forage maize leaves, stems and grains was placed in a test tube, 10 mL of phosphate buffer (0.1 mol L^−1^, pH 7.5) was added, and the test tube was placed in darkness for 20 min. Afterward, 1 mL of 30% trichloroacetic acid was added and then immediately oscillated. A portion of the extract (2 mL) was transferred into a test tube, and 4 mL of 1% sulfonamide and 4 mL of 0.02% α-naphthylamine solutions were added. The sample solution was shaken thoroughly and allowed to stand for 35 min. Finally, absorbance was measured at 520 nm using a spectrophotometer (UV-4802S, Shanghai Chongfen Scientific Instrument Co., Ltd., Shanghai, China).

Measurement of glutamine synthetase (GS) activity: Fresh forage maize leaves, stems and grains of 0.5 g fresh weight were ground to powder and placed in an ice bath with 0.05 mol L^−1^ Tris-HCl extraction buffer (containing 2 mmol L^−1^ MgSO_4_, 2 mmol L^−1^ DTT, and 0.4 mol L^−1^ sucrose at pH 8.0). Subsequently, the sample extracts were centrifuged for 20 min at 15,000× *g* and 4 °C. After centrifugation, the supernatant containing the GS crude extract was collected. The absorbance was measured using a spectrophotometer at 540 nm (UV-4802S, Shanghai Chongfen Scientific Instrument Co., Ltd., Shanghai, China), and GS activity was expressed as the formation of γ-glutamylhydroxamate at 37 °C.

Measurement of glutamic pyruvic transaminase (GPT) activity: Fresh forage maize leaves, stems and grains of 0.5 g fresh weight were ground to powder and placed in an ice bath with 0.05 mol L^−1^ Tris-HCl extraction buffer (containing 0.2 mol L^−1^ trimethylolaminomethane [50 mL] and 0.2 mol L^−1^ HCl [44.2 mL] diluted with distilled water to 200 mL at pH 7.2). Thereafter, the sample extracts were centrifuged for 20 min at 20,000× *g* and 4 °C. The OD value of the supernatant containing the crude enzyme extract was measured at 505 nm using a spectrophotometer (UV-4802S, Shanghai Chongfen Scientific Instrument Co., Ltd., Shanghai, China), and GPT activity was expressed as the catalytic production of 1 nmol pyruvate per minute.

Measurement of crude protein content: Forage maize leaves, stems and grains were dried at 105 °C in an electric blast drying oven (DHG-9070A; Beijing Hongda Tianju Test Equipment Co., Ltd., Beijing, China) to fix the chlorophyll and then dried at 80 °C to a constant weight and crushed. The crushed samples were passed through a 40-mesh sieve, and crude protein content was determined using the Kjeldahl method.

Partial Factor Productivity of N (PFPTN) and K (PFPTk): The calculation formula is as follows: *PFP_TN/Tk_ = Y_d_/F*; in the formula, *Y_d_* is the yield of crude protein yield per unit area (Kg · ha^−1^), and *F* is the nitrogen/phosphorus application rate.

### 2.5. Statistical Analysis

The SAS 8.0 (SAS Institute Inc., Cary, NC, USA) was used to analyze variance, the least significant difference test (*p* < 0.05), and correlation analysis to determine the differences and correlation degree in chemical fertilizer placement, N/K, GS activity, NR activity, GPT activity and crude protein content in leaves, stems, and grains.

## 3. Results

### 3.1. Rainfall and Average Temperature During the Experimental Period

**Figure 2:**
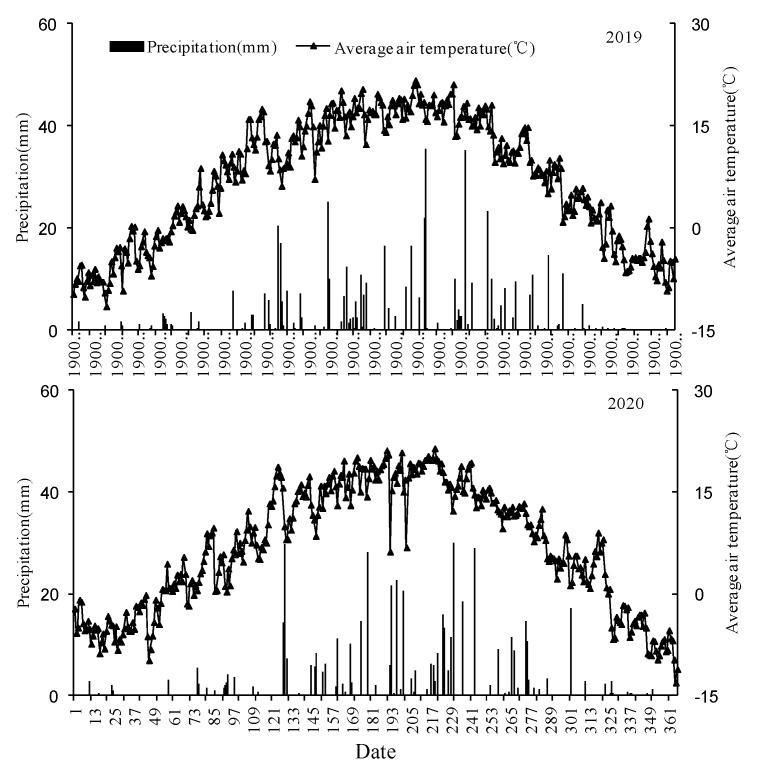
Precipitation and air temperature during the 2019–2020 periods.

Based on the meteorological data obtained from the Dingxi Experimental Station at the Gansu Academy of Agricultural Sciences, the annual rainfall of the study area in 2019 was 516.8 mm, and the rainfall received during the forage maize growth period was 358.8 mm, which was a humid–wet year. The amount of rainfall received in 2020 was 518.9 mm, and the amount of rainfall received during the forage maize growth period was 386.2 mm, which was a humid–wet year. The annual average temperature in 2019 was 7.2 °C, the average temperature during the crop growth period was 15.9 °C, and the annual average temperature in 2020 was 6.9 °C. The average temperature during the crop growth period was 15.6 °C (Figure 2).

### 3.2. Effect of Chemical Fertilizer Placement on Soil Average Temperature and Water Storage

**Figure 3:**
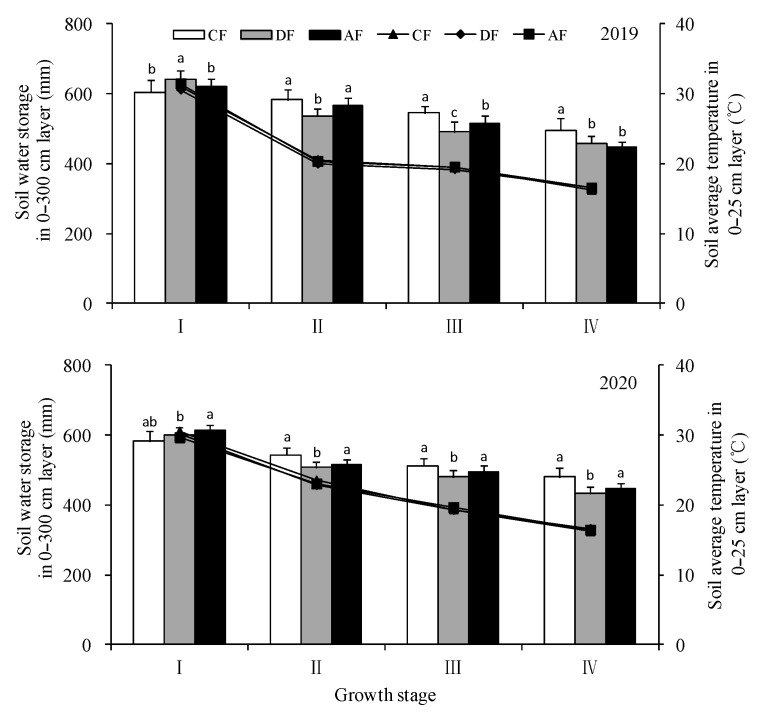
Effect of fertilization depth on soil water storage in the 0–300 cm layer and average temperature in the 0–25 cm layer. Notes: The bar represented soil water storage in the 0–300 cm layer, and the line represented soil average temperature in the 0–25 cm layer. I: jointing stage, II: tasseling stage, III: filling stage, IV: dough stage. The following I, II, III, IV is the same means. Different letter on the bars means the significant difference with confidence level set at 0.05, the same below. Error bars represent standard error (n = 3).

Soil temperature is mainly related to the coverage; all treatments used double ridges and furrows with plastic mulching in the experiment; therefore, the soil temperature in the 0–25 cm layer is basically the same under different fertilization depths. Soil water storage in the 0–300 cm layer at different growth stages was related to crop growth, and there were significant differences among treatments (Figure 3). With the advancement of the maize growth period, the water consumption of the aboveground part of the maize increased; therefore, the soil water storage gradually decreased. At the tasseling, filling and dough stage of maize, the soil water storage in the 0–300 cm layer in DF was significantly lower than that of CF and AF, which may be related to the vigorous growth of aboveground plants in DF and the greater consumption of soil moisture.

### 3.3. Effect of Chemical Fertilizer Placement on Content of Total N and Total K in Different Forage Maize Organs

**Figure 4:**
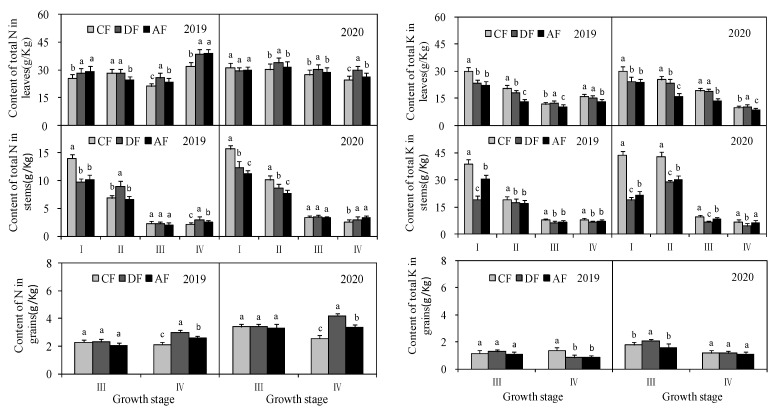
Effect of fertilization depth on content of total N and total K in different forage maize organs.

Chemical fertilizer placement had a significant effect (*p* < 0.05) on the content of total N in different forage maize organs (Figure 4). Comprehensive analysis of two years of data shows deep fertilization could significantly increase the content of total N in leaves, stems and grains at the dough stage. The content of total N in leaves, stems and grains increased on average by 20.59%, 29.64% and 53.06% in DF, respectively, and by 13.88%, 26.73% and 26.79% in AF, respectively, when compared to that of CF. The content of total K varied with the growth period and planting years of forage maize. The effect of chemical fertilizer placement on the content of total K did not have an obvious regular pattern.

### 3.4. Effect of Chemical Fertilizer Placement on N/K Ratios in Different Forage Maize Organs

**Figure 5:**
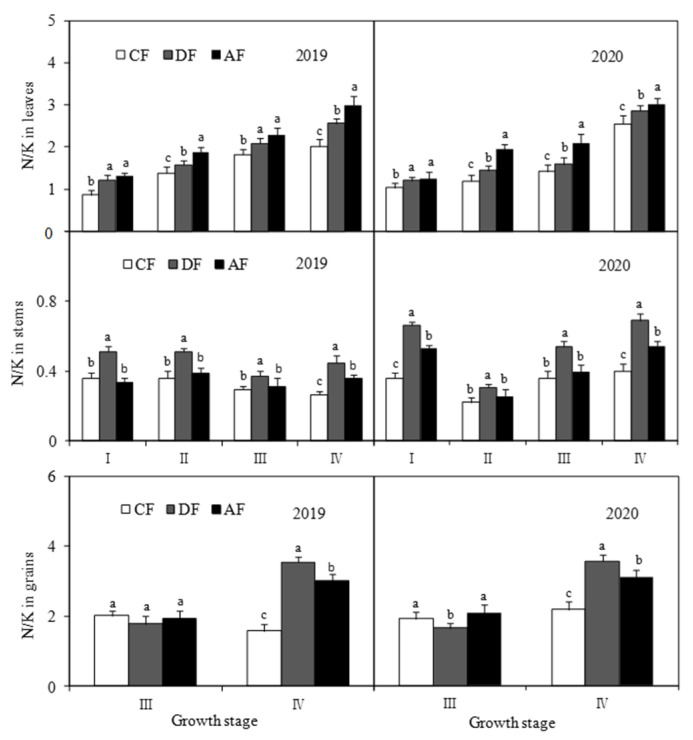
Effect of fertilization depth on nitrogen/potassium (N/K) ratios in different forage maize organs. Notes: I: jointing stage, II: tasseling stage, III: filling stage, IV: dough stage. The following I, II, III, IV is the same means.

Chemical fertilizer placement had a significant effect (*p* < 0.05) on the N/K ratio in different forage maize organs (Figure 5). DF and AF significantly increased (*p* < 0.05) the leaf N/K ratio of forage maize at each growth stage. The N/K ratios of forage maize leaves at jointing, tasseling, filling, and dough stages increased by 28.69%, 17.34%, 13.59%, and 19.51% in DF, respectively, and by 36.64%, 49.03%, 36.48%, and 33.01% in AF, respectively, when compared to those in CF. DF significantly increased (*p* < 0.05) N/K ratios of forage maize stems, with average increases of 63.26%, 40.31%, 37.89%, and 69.94% being observed at jointing, tasseling, filling, and dough stages, respectively. The N/K ratios of forage maize grains in DF and AF were 108.95% and 53.15% higher than those in CF.

### 3.5. Effect of Fertilization Depth on NR Activity in Different Forage Maize Organs

**Figure 6:**
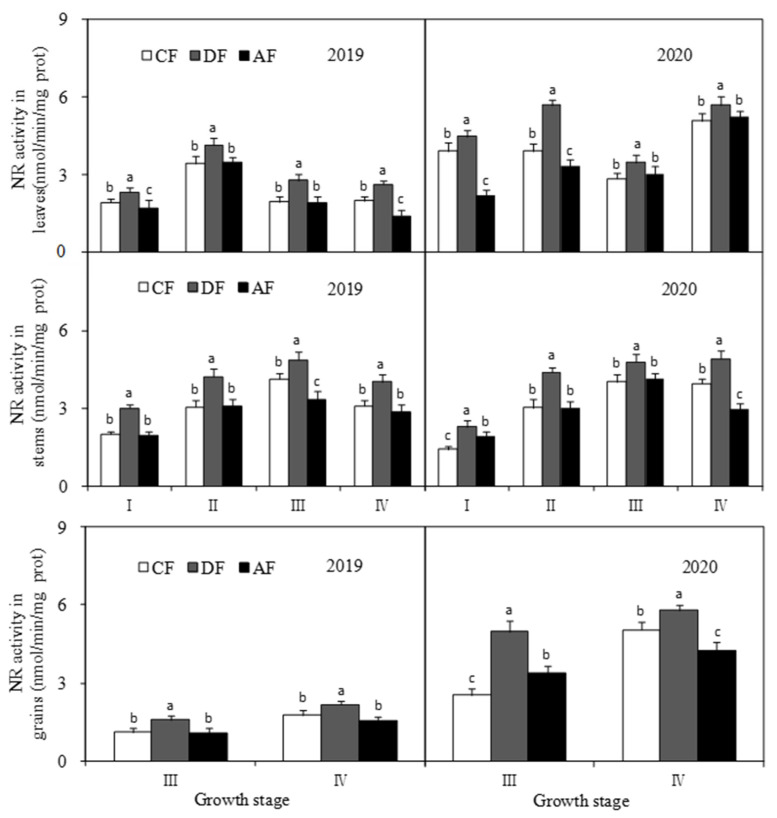
Effect of fertilization depth on nitrate reductase (NR) activity on different forage maize organs.

DF had a significant effect (*p* < 0.05) on NR activity in forage maize leaves, stems, and grains (Figure 6). NR activity of forage maize leaves at jointing, tasseling, filling, and dough stages increased by 17.25%, 33.36%, 32.56%, and 22.02% when compared to that of CF and by 68.67%, 45.54%, 30.83%, and 71.84% when compared to that of AF, with significant differences being observed between treatments. NR activity of forage maize stems in DF at jointing, tasseling, filling, and dough stages was significantly higher (*p* < 0.05) by 55.53%, 41.34%, 18.19%, and 27.17% than that in CF and significantly higher by 35.60%, 41.18%, 30.72%, and 65.56% than that in AF, respectively. NR activity of forage maize grains in DF at the filling and dough stages was significantly higher (*p* < 0.05) than that in CF and AF, which increased by 68.60% and 19.35% at the grain filling stage and by 45.99% and 37.53% at the dough stage, respectively. The trends exhibited by NR activity in forage maize grains in CF and AF varied in different planting years and at different growth stages.

### 3.6. Effect of Fertilization Depth on GS Activity in Different Forage Maize Organs

**Figure 7:**
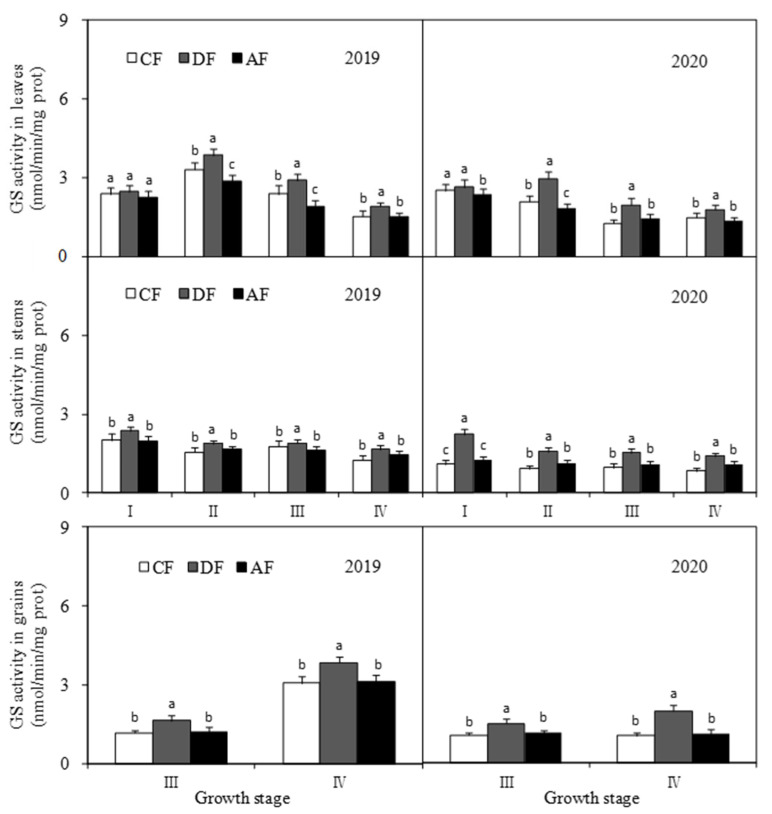
Effect of fertilization depth on glutamine synthetase (GS) activity on different forage maize organs.

Chemical fertilizer placement had varying effects on GS activity in different forage maize organs (Figure 7). The GS activity of forage maize leaves in DF at the tasseling, filling, and dough stages was 28.49%, 38.30%, and 22.20%, significantly higher (*p* < 0.05) than that in CF, and 46.98%, 44.32%, and 28.87% significantly higher than that in AF, respectively. Significant differences (*p* < 0.05) were observed in GS activity between CF and AF due to the growth period and planting year. Specifically, the GS activity of forage maize leaves in CF was significantly higher (*p* < 0.05) than that in AF at the tasseling and filling stages in 2019, and at the jointing and filling stages in 2020.

The GS activity of forage maize stems in DF was significantly higher (*p* < 0.05) than that in CF and AF, and no significant difference (*p* < 0.05) was observed between CF and AF. The GS activity of forage maize stems in DF at the jointing, tasseling, filling, and dough stages was 61.26%, 45.58%, 33.74%, and 51.26% higher than that in CF, respectively, and 49.50%, 25.92%, 28.26%, and 22.35% higher than that in AF, respectively.

The GS activity of forage maize grains in DF was significantly higher (*p* < 0.05) than that in CF and AF, and no significant difference (*p* < 0.05) was observed between CF and AF. Specifically, the GS activity of forage maize grains in DF at the filling and dough stages increased by 38.97% and 55.95% when compared to that in CF and by 33.32% and 47.93% when compared to that in AF, respectively.

### 3.7. Effect of Fertilization Depth on GPT Activity in Different Forage Maize Organs

**Figure 8:**
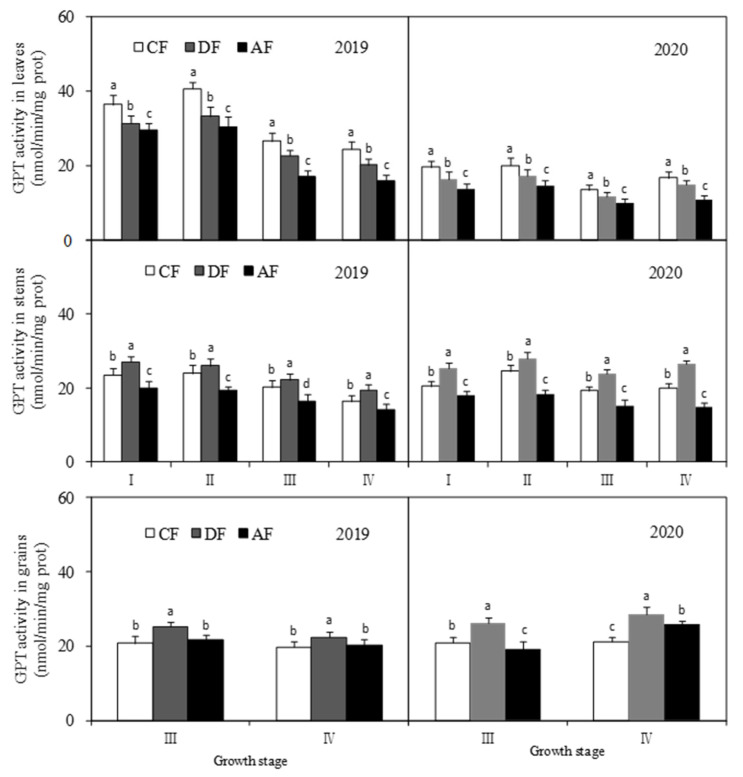
Effect of fertilization depth on glutamic pyruvic transaminase (GPT) activity in different forage maize organs.

The GPT activity of forage maize leaves at different fertilization depths was in the order of CF > DF > AF, and the differences between treatments were significant (*p* < 0.05) (Figure 8). GPT activity of forage maize leaves in CF at the jointing, tasseling, filling, and dough stages was 17.55%, 19.19%, 16.24%, and 16.80% higher than that in DF, respectively, and 24.46%, 26.19%, 30.89%, and 35.23% higher than that in AF, respectively. GPT activity of forage maize stems among the treatments was in the order of DF > CF > AF, and the differences between treatments were significant (*p* < 0.05). Specifically, the GPT activity of forage maize stems in DF at the jointing, tasseling, filling, and dough stages was 19.66%, 10.96%, 17.02%, and 25.29% higher than that in CF, respectively, and 38.57%, 43.01%, 46.12%, and 57.28% higher than that in AF, respectively. DF significantly increased (*p* < 0.05) GPT activity at the grain filling and dough stages. The GPT activity of forage maize grains in DF was 23.63% and 23.95% on average higher than that in CF and 25.76% and 11.05% on average higher than that in AF, respectively.

### 3.8. Crude Protein Content in Different Forage Maize Organs at Different Fertilization Depths

**Figure 9:**
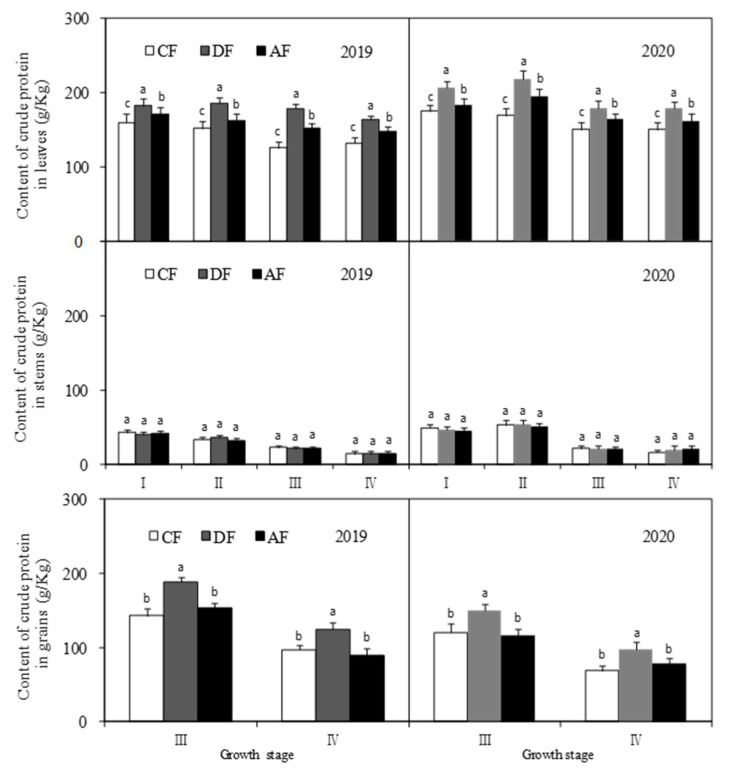
Content of crude protein in different organs of forage maize.

The crude protein content of leaves among the treatments was in the order of DF > AF > CF, and the differences between treatments were significant (*p* < 0.05) (Figure 9). The crude protein contents of forage maize leaves in DF at the jointing, tasseling, filling, and dough stages increased by 9.88%, 13.05%, 13.49%, and 10.41%, respectively, when compared to those in AF and by 16.12%, 25.94%, 30.54%, and 20.99%, respectively, when compared to those in CF. No significant differences (*p* < 0.05) were observed in the crude protein content of forage maize stems among the treatments. The crude protein content of forage maize grains in DF was significantly higher (*p* < 0.05) than that in other treatments, and no significant differences were observed between AF and CF. The crude protein content of grains in DF at the grain filling and dough stages increased by 25.91% and 32.12%, respectively, when compared to that in AF and by 28.12% and 35.39%, respectively, when compared to that in CF.

### 3.9. Correlations Between Activities of Key Enzymes Associated with Crude Protein Synthesis and Crude Protein Content in Forage Maize

**Figure 10:**
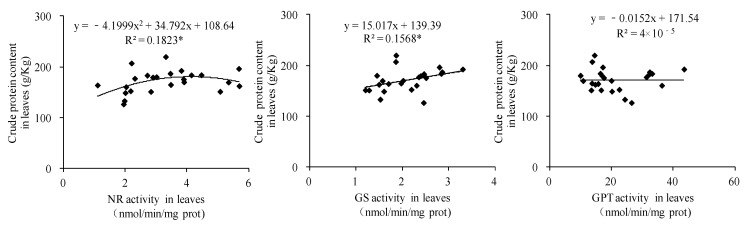
Correlations between the activities of key enzymes associated with crude protein synthesis and crude protein content in forage maize leaves. Note: “*” and “**” indicate the significant correlation at the 0.05 and 0.01 probability level, respectively. The dots represent the measured values, and the lines represent the correlation trend in the figure, the same below.

**Figure 11:**
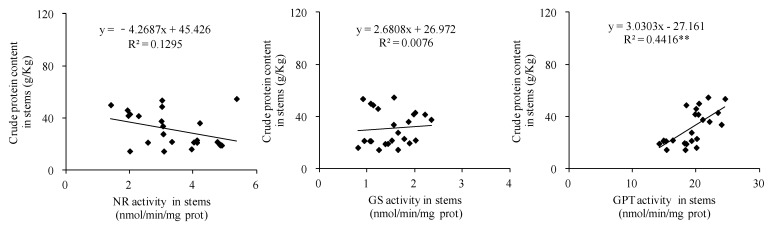
Correlations between the activities of key enzymes associated with crude protein synthesis and crude protein content in forage maize stems.

**Figure 12:**
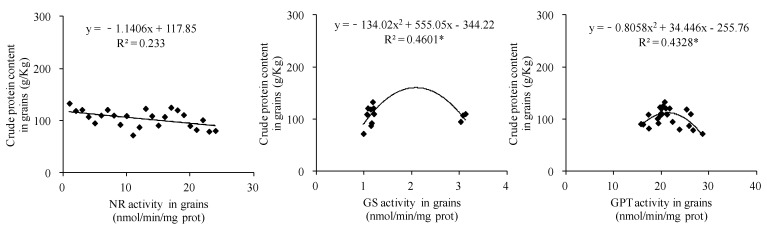
Correlations between the activities of key enzymes associated with crude protein synthesis and crude protein content in forage maize grains.

Comprehensive analysis of two years of data revealed a significant quadratic parabolic relationship between the crude protein content and NR activity in leaves (R^2^ = 0.1823 *). Crude protein content was positively correlated with GS activity in forage maize leaves (R^2^ = 0.1568 *), but had no significant correlation with GPT activity in leaves (Figure 10). Crude protein content was significantly positively correlated with GPT activity in forage maize stems (R^2^ = 0.4416 **) but had no significant correlation with NR and GS activities in forage maize stems (Figure 11). A significant quadratic parabolic relationship was observed between crude protein content and GS and GPT activities in forage maize grains (R^2^ = 0.4601 * and 0.4328 *); however, no significant correlation was observed between crude protein content and NR activity in forage maize grains (Figure 12).

### 3.10. Partial Factor Productivity of N (PFP_TN_) and K (PFP_Tk_) at Different Fertilization Depths

Chemical fertilizer placement had a significant effect (*p* < 0.05) on the content of total N in different forage maize organs (Table 1). In 2019, PFP_TN_ and PFP_TK_ in DF increased by 18.71% and 18.71%, respectively, when compared to that of CF, and by 14.83% and 14.83%, respectively, when compared to that of AF; all differences were significant (*p* < 0.05). There is no significant difference between CF and AF. In 2020, PFP_TN_ and PFP_TK_ were in order of DF > AF > CF, and the difference between treatments was significant (*p* < 0.05). Among them, PFP_TN_ and PFP_TK_ in DF are 65.43% and 65.43% higher than that of CF and 36.54% and 36.54% higher than that of AF.

## 4. Discussion

### 4.1. Chemical Fertilizer Placement Significantly Affected Crude Protein Synthesis in Forage Maize

Deep fertilization can prolong nitrogen supply duration in the soil [24], meet the nutrient demand of crops during the whole growth period [25], increase nitrogen, phosphorus, and potassium in plants, delay leaf senescence, maintain a high green leaf area, increase the net photosynthetic rate [26,27], and considerably improve nitrogen uptake and utilization rate in wheat, corn, potatoes, and other crops [13,14,20]. As an important component of enzymes, nucleic acids, and proteins, nitrogen plays a crucial regulatory role in crop nitrogen metabolism, activities of key enzymes associated with nitrogen metabolism, amino acid synthesis and transformation, and protein synthesis [23].

In this study, chemical fertilizer placement had a significant effect on crude protein synthesis in forage maize, and the application of nitrogen fertilizer considerably influenced crude protein content in leaves and grains (Figure 9). DF significantly increased the crude protein content of forage maize leaves at the jointing, tasseling, filling, and dough stages (Figure 9). The crude protein content of forage maize grains in DF also increased significantly. The results indicate that chemical fertilizer placement had a significant effect on crude protein synthesis in forage maize and fertilization at a depth of 30 cm (DF) significantly increased the crude protein content of forage maize and improved Partial Factor Productivity of N (PFP_TN_) and K (PFP_Tk_) (Table 1). Most of the existing research on deep application of chemical fertilizers has focused on crop growth and soil nutrients [13,20,24,28]. This study is a relatively new discovery; on one hand, this discovery enriches the research content of deep application of chemical fertilizers; on the other, it provides new ideas for crop quality regulation in the future.

### 4.2. Chemical Fertilizer Placement Significantly Regulated the Activities of Key Enzymes Associated with Crude Protein Synthesis in Forage Maize

The level of N metabolism is a key indicator of crude protein synthesis ability and forage maize yield. Enzyme activity is a major factor that influences nitrogen metabolism. NR, which largely occurs in plant leaves, is a key enzyme associated with nitrate assimilation during nitrogen metabolism. NR activity has been shown to be positively correlated with grain protein content and is sensitive to external nitrogen fertilizer [29]. GS is another key rate-limiting enzyme involved in nitrogen metabolism and is crucial in regulating crop growth and yield. GPT is one of the most important transaminases in plants, and its activity can reflect the effect of amino transfer in plant nitrogen metabolism. The activities of NR, GS, and GPT were closely related to protein synthesis and were significantly affected by the amount, type, and nitrogen fertilizer application period [30,31]. A previous study revealed that the activities of key enzymes associated with nitrogen metabolism increased with an increase in nitrogen levels, which in turn increased grain protein content and yield [32]. Layered deep application of nitrogen fertilizer can promote nitrogen absorption and improve the utilization efficiency of nitrogen fertilizer [33], which subsequently influences the activities of key enzymes associated with crude protein synthesis. In this study, NR and GS activities in forage maize leaves, stems, and grains in DF increased significantly (Figure 6 and Figure 7). GPT activity in forage maize leaves was in the order of CF > DF > AF and was significantly higher in stems and grains in DF. The results suggest that deep application of fertilizer at 30 cm and average fertilizer application at depths of 15 cm and 30 cm significantly improved the activities of key enzymes associated with crude protein synthesis and then substantially promoted crude protein synthesis in forage maize, which was consistent with the existing study [29,30,31,32].

### 4.3. Chemical Fertilizer Placement Improved Crude Protein Synthesis by Increasing the Activities of Key Enzymes Associated with Protein Synthesis

Appropriate fertilization depth can increase nitrogen, phosphorus, and potassium contents in plants; increase the activities of superoxide dismutase, peroxidase, and catalase in plant leaves; delay leaf senescence; increase net photosynthetic rate, water and fertilizer use efficiency; and substantially increase crop yield [26,27,34]. Several studies have shown that deep fertilization can promote nutrient absorption by maize plants, significantly increase the nitrogen content of leaves, and improve nutrient transport and assimilation capacity of vegetative organs [28,35]. The relatively high nitrogen content in leaves provides additional energy and electrons for amino acid synthesis [36] and increases free amino acid contents and the activities of key enzymes (NR, GS, and GPT) associated with protein synthesis in plant organs [15]. The activity of key enzymes involved in protein synthesis is significantly correlated with crude protein content [20]; therefore, an appropriate fertilization depth promotes protein synthesis, which was confirmed by the findings of this study.

In this study, DF significantly increased the N/K ratio in forage maize leaves, stems and grains (Figure 5) and improved nutrient transport and assimilation capacity of vegetative organs. Moreover, DF practice significantly impacts the activities of key enzymes associated with protein synthesis (Figure 6, Figure 7 and Figure 8), which was consistent with the existing study [25,32]. Under the premise of the increase in the nitrogen content in each organ of forage maize, the improvement of the activities of key enzymes associated with protein synthesis, and the enhancement of correlation between enzyme activity and crude protein content (Figure 10, Figure 11 and Figure 12), DF significantly increased crude protein content in forage maize leaves by 11.71–23.40% and in forage maize grains by 29.02–45.82%. The results indicate that layered deep application of chemical fertilizer increased the crude protein content by enhancing the activities of key enzymes associated with crude protein synthesis in forage maize organs. This is a new research result, and there are very few similar reports.

## 5. Conclusions

Chemical fertilizer placement exerted significant effects on forage maize N/K ratios, activities of key enzymes associated with crude protein synthesis, and crude protein content. The N/K ratio, NR and GS activities of leaves, stems and grains, as well as GPT activity in stems and grains, increased significantly with an increase in the fertilization depth. Significant positive correlations were observed between crude protein content and NR and GS activities in leaves, crude protein content and GPT activity in stems, and crude protein content and GS and GPT activities in grains. DF significantly increased crude protein content in forage maize leaves (11.71–23.40%) and grains (29.02–45.82%) by improving the whole plant N/K ratio, NR and GS activities in leaves, and GS and GPT activities in grains. These results clarified the regulation mechanism of deep application of chemical fertilizer on crude protein synthesis of forage maize, and the deep application of chemical fertilizer will become a new and effective way to regulate the quality of forage maize. Fertilizer placements using innovative machines and GPS tools for precision farming in order to maximize outputs for both agronomists and animal husbandry projects/silage makers.

## Figures and Tables

**Table 1:** Effect of fertilization depth on Partial Factor Productivity of N (PFP_TN_) and K (PFP_Tk_).

Year	Treatment	Yield of Crude Protein (Kg ha^−1^)	Nitrogen Rate (Kg ha^−1^)	PFP_TN_ (Kg kg^−1^)	Potassium Rate (Kg ha^−1^)	PFP_Tk_ (Kg ha^−1^)
2019	CF	5917.22 b	300.00	19.72b	135.00	43.83 b
	DF	7024.46 a	300.00	23.41a	135.00	52.03 a
	AF	6117.23 b	300.00	20.39b	135.00	45.31 b
	CF	5393.56 c	300.00	17.98c	135.00	39.95 c
2020	DF	8922.81 a	300.00	29.74a	135.00	66.09 a
	AF	6535.06 b	300.00	21.78b	135.00	48.41 b

Note: Different letter in the table means the significant difference with confidence level set at 0.05.

## Data Availability

Dates are contained within article.
